# Prevalence and determinants of household solid waste management practice among residents of the city corporations of Dhaka, Bangladesh

**DOI:** 10.1371/journal.pone.0346265

**Published:** 2026-03-27

**Authors:** Irsat Jahan, Kh. Farhad Hossain, Syed Sharaf Ahmed Chowdhury, Afnan Mohd Shafiullah, Sadia Shafi, Azaz Bin Sharif

**Affiliations:** Department of Public Health, North South University, Dhaka, Bangladesh; Cranfield University, UNITED KINGDOM OF GREAT BRITAIN AND NORTHERN IRELAND

## Abstract

Solid waste management (SWM) has emerged as a global concern, posing critical environmental and public health challenges. Dhaka’s ranking as the second most polluted city in the world between 2018 and 2021 highlights the need for a more sustainable waste management system. Household solid waste is mismanaged, as evident from illegal dumping in unauthorized places, scattered garbage and overflowing waste bins on the roads, and clogged drains creating severe environmental health hazards. This study aimed to identify the prevalence and determinants of household SWM practices among residents of the city corporations of Dhaka, Bangladesh. A cross-sectional survey was conducted from June to July 2025 among 496 residents using a pre-tested structured questionnaire administered through face-to-face interviews. Descriptive statistics were used to summarise participants background characteristics and their levels of knowledge, perception, and practice related to household SWM. Associations between independent variables and SWM practice were examined using chi-square tests, and multivariable binary logistic regression was performed to determine potential factors associated with good practice. The prevalence of good household SWM practice was 47.18%. Knowledge (AOR = 1.23; 95% CI: 1.12–1.35), perception (AOR = 1.14; 95% CI: 1.06–1.22), and exposure to information regarding solid waste management (AOR = 1.91; 95% CI: 1.27–2.89) were significantly associated with good practice. Overall, the findings indicate that household SWM practices among residents of the city corporations of Dhaka remain suboptimal. Increasing access to SWM-related information through targeted communication strategies may enhance household-level SWM practices.

## Introduction

Solid waste management (SWM) has emerged as one of the most pressing environmental and public health challenges worldwide, particularly in rapidly developing nations [1]. The growing volume of solid waste in urban centres has overwhelmed municipal systems, leading to environmental degradation, water and air pollution, and increased risk of disease transmission [2,3]. Southeast Asian cities, in particular, have faced escalating waste management crises over recent decades as a result of rapid urbanization and industrial growth [2]. Dhaka, the capital of Bangladesh, exemplifies these challenges [4]. Once a small urban centre, it has transformed into a megacity driven by globalization, industrialization, and rural-to-urban migration [2,4]. This expansion, coupled with rising consumerism and economic growth, has led to an exponential increase in waste generation, turning inadequate waste management into a critical public health issue [3].

Dhaka is one of the most densely populated cities in South Asia, with a population exceeding 24 million and growing steadily due to internal migration [5]. Unplanned urbanization, limited infrastructure, and poor governance have compounded the problem [6]. The city produces approximately 6,500 tons of solid waste per day, estimated to outreach 8,500 tons by the year of 2032 [7]. Shockingly 40–60% of waste remains uncollected due to poor infrastructure [8], accumulating in open spaces, drains, and water bodies or is burned in the open, releasing hazardous pollutants [9]. The underlying reason is that existing landfills are nearing full capacity, and establishing new dumping sites has become increasingly difficult due to high land costs and limited land availability [10,11]. Consequently, uncollected waste contributes to environmental contamination, foul odours, and the proliferation of disease vectors, exacerbating public health risks [11]. For instance, the sharp rise in dengue cases in Dhaka has been linked to waste materials such as discarded plastics and containers that serve as mosquito breeding sites [9]. Despite the presence of two city corporations responsible for waste collection and disposal, inefficiencies in transportation, inadequate resource allocation, and limited financial capacity continue to impede effective management [12]. Dhaka’s ranking as the second most polluted city in the world during 2018–2021 underscores the urgency of implementing stronger, more sustainable waste management systems [13].

Despite the existence of the National 3R Strategy for Waste Management, which provides national guidance on waste reduction, reuse, and recycling [14], and the legally enforceable Solid Waste Management Rules, 2021, which provide regulations for SWM responsibilities targeting households, city corporations and service providers [15], implementation gaps remain. These gaps arise from obstacles at multiple stages of execution, including waste generation, collection and transportation, disposal, recycling and treatment, as well as monitoring, regulation, and governance [8], which help explain why SWM practices remain suboptimal. This gap also reflects insufficient coordination between government institutions and citizens, as well as the absence of sustained community engagement mechanisms [16]. At the household level, solid waste collection in Dhaka City remains heavily dependent on city corporation services, with minimal community participation [8]. The urban structure of Dhaka, characterized by high population density [17], compact apartment-based housing [18], predominantly rented households [19], and relatively small household sizes [20], further constrains household SWM practices. Household waste segregation and recycling practices are rare, largely because there is limited indoor space, and policies regarding mandatory waste separation at sources are not enforced [8] instead, unsegregated waste is collected by the city corporation’s waste collectors [8,9]. Thus, urban households under the city corporations of Dhaka may have poor SWM practices due to such structural barriers. In addition, SWM practices can be influenced by domestic helpers who routinely assist with household chores, including waste management, particularly in urban households with middle- and higher-income residents [21]. Promoting information, such as guidance or knowledge on proper household SWM practices, including waste sorting, separation, disposal methods, and the principles of reduce, reuse, and recycle (3Rs), through various channels such as social media, local newspapers, radio, television, brochures, or announcements by the local government [22], can significantly increase people’s awareness and promote good SWM habits [23].

Evidence from Malaysia [24], Thailand [22], Ethiopia [25–28], and Ghana [29] suggests that insufficient knowledge and poor perception among residents lead to poor waste management practices. Therefore, effective household waste management practices require sufficient public knowledge, positive perception, behavioural change, and effective participation [10,24]. Promoting local initiatives and global best practices of solid waste management at the household level has shown not only the reduction of environmental burden but also enhanced community empowerment and shared responsibility toward a cleaner and healthier city [30]. Thus, understanding citizens’ knowledge, perception, and practices regarding household SWM practice is fundamental for developing effective interventions and sustainable urban policies [30,31].

In Bangladesh, most studies on solid waste management have focused on municipal [32,33] and city corporation operations [9,34], recycling market mechanisms [16], policy implementation and its impact [8,16], institutional limitations [8], resource recovery [9], with comparatively little attention to household-level behaviour. A few studies have assessed knowledge and practices but not perceptions among specific groups, such as students [31], underprivileged children [35], or residents of selected urban areas [36]. These studies have been geographically and demographically narrow in scope and limited in methodological approach, as some adopted a qualitative approach while others examined associations between socio-demographic factors and practice, which may have limited the detection of independent associations of knowledge and perception with household SWM practices in a larger urban population [31,35,36]. Addressing this gap, the present study employs a larger, more diverse urban sample along with multivariable regression methods to better examine these relationships. The aim of this study is to assess the prevalence of household SWM practice and identify its associated determinants among residents of the city corporations of Dhaka, Bangladesh. By focusing on the household dimension of waste management, this study will provide essential insights for designing community-based strategies and policies to strengthen sustainable waste management systems in Bangladesh.

## Methods

### Study design and participant selection

A cross-sectional study design was used to assess the prevalence and determinants of household solid waste management (SWM) practices among the residents of the two city corporations of Dhaka, namely Dhaka North City Corporation (DNCC) and Dhaka South City Corporation (DSCC) from 01 June 2025–30 July 2025.

The study participants were permanent residents of both North and South City Corporation of Dhaka. The participants were selected conveniently from different households who were residing in formal residential buildings. Only one participant from each building was included and participants from adjacent buildings were avoided to make sure that broader and different areas could be covered. As convenient sampling is a non-probability sampling method, the sample may not be representative of the broader population, and the generalizability of the findings should therefore be interpreted with caution.

Waste management at the household level is predominantly handled by women in the study setting [37]. Although some men may also participate, this study focused only on women who were primarily responsible for household SWM practices; thus, men were excluded from the sample. Women living alone without families were also excluded. Our inclusion criteria were women aged 18 years or older. This criterion included both married women and unmarried women living with their parents, provided they were the responsible personnel for managing their household solid waste. The study included participants living in formal residential buildings, defined as houses or apartment buildings with access to door-to-door waste collection services provided by the DNCC and DSCC. Residents from slum areas in both city corporations were excluded. Slums are densely packed with informal housing structures and narrow roads, which limit the accessibility of door-to-door waste collection services. Consequently, the exclusion of slum areas, combined with the convenient selection of study participants, limits the generalizability of the findings to all households in the city corporations of Dhaka.

### Study site

Dhaka is the capital city of Bangladesh and is divided into two city corporations: Dhaka North City Corporation (DNCC) and Dhaka South City Corporation (DSCC). DNCC spreads over an area of 196.22 km² with 54 wards, and DSCC spreads over an area of 109.24 km² with 75 wards. DNCC has a population of 5,979,537; on the contrary, DSCC has a population of 4,299,345 [20]. Formal buildings are the predominant housing types, followed by slums or informal settlements.

Both city corporations are responsible for collecting household waste from their respective wards. The study included areas from both corporations regardless of socioeconomic status. Household waste collection is divided into two parts, namely primary collection and secondary collection [38]. Primary collection is where waste generation points, i.e., household residents, are in charge of taking their household waste to the city corporation’s waste collection points, such as large dustbins or containers located at each building or residence [38]. Different organizations contract with both city corporations to serve as door-to-door waste collectors [39]. They are called Primary Collection Service Providers (PCSP) as they collect waste from the household’s waste collection points [38]. They carry the waste through rickshaw vans, a small three-wheeled vehicle, to the secondary collection points, which are large containers located at designated places, and from there the waste is transported by large collection trucks to the landfill sites [38,39]. The whole waste collection process is carried out in an unsegregated manner [39]. Thus, an informal market consisting of scavengers, locally known as tokai, and hawkers play a significant role in recycling solid waste [39,40]. Tokais gather and pile up recyclable items from waste bins located at secondary collection points and landfill sites, while hawkers buy such recyclable items door-to-door from households of different areas [39–41]. Both groups trade the recyclable items to recycle waste dealers, locally known as bhangari [39]. The bhangaris clean, wash, dry and sort the recyclables to trade them again in the market [39].

Waste collection services are generally better in wider roads and commercial areas, where large vehicles can operate efficiently. In narrow roads and slum areas, large vehicles often cannot reach or may create roadblocks, limiting accessibility. Thus, waste collection frequency varies across areas. Only formal buildings have access to daily door-to-door waste collection services provided by the city corporations [42]. Waste collection in slum households is different, as no door-to-door services are provided daily due to restricted access and lack of formal recognition [41,43]. Slum residents typically discard waste at nearby secondary collection points, where collection trucks infrequently transport it to landfills.

### Sample size

No study has been conducted in Bangladesh at the household level to establish the prevalence of household SWM practice. Based on a previous study conducted among school students in Bangladesh, where the prevalence of solid waste management practice was reported as 51.12% (p = 0.51) [31], the required sample size for our study was calculated using the formula n = *z^2^pq/d^2^* with a 95% confidence level (z = 1.96) and a 5% margin of error (d = 0.05). By substituting the values (z = 1.96, p = 0.51, q = 0.49, and d = 0.05) into the formula, the minimum required sample size was calculated to be 385 (rounded). However, a larger sample of 496 participants was collected.

### Data collection process and instruments

Data were collected using a pre-tested structured questionnaire through face-to-face interviews with the participants. The questionnaire was developed based on a thorough literature review [25,26,32,36] and various guidelines on the 3R (Reduce, Reuse and Recycle) strategy [44,45] for waste management, which were later modified according to the study context. The questionnaire was prepared in English and later on, it was translated to Bangla. Data collection was conducted by trained data collectors and the questionnaire was pre-tested before the actual collection with a small sample size of 30. Based on the feedback from the pre-test, the questionnaire was revised and edited to make the instructions, questions, and responses more understandable. The questionnaire consisted of closed-ended questions on background information and participants’ knowledge, perception, and practice of household solid waste management. Background information included participants’ age, family size, highest education level, husband’s highest education level, employment status, monthly household income reported in Bangladeshi Taka (BDT), residential status, and exposure to SWM information. Exposure to SWM information was defined as receiving any guidance or knowledge on proper household SWM practices, including waste sorting, separation, disposal methods, and the principles of 3Rs, through multiple channels, including social media, radio or television, newspapers, posters or flyers, and announcements by the local government.

Participants’ knowledge of household SWM was assessed using 12 different questions with binary response (yes or no) options where 1 was coded as “yes” and 0 was coded as “no”. The knowledge score ranged from 0 to 12. The higher the score the better is the knowledge. Participants’ perception of household SWM was assessed using 7 questions. Each item was measured on a four-point Likert scale ranging from strongly agree (1) to strongly disagree (4). All the 7 questions were reverse coded. The questions are: “Do you think purchasing products with minimal packaging can reduce the amount of waste?”, “Do you think digitalization can reduce the amount of solid waste?”, “Do you think re-using the reusable products can reduce the solid waste?”, “Do you think improper waste management can cause health problems?”, “Do you think individuals from the society should be a part of proper waste management?”, “Do you think sorting solid waste in the household can lead to proper solid waste management in the city?”, “Do you think solid waste management is a problem in your area?”. Regarding the question on digitalization, it was clarified that it referred to the use of digital food delivery apps that deliver groceries in paper bags, cardboard boxes, or recyclable bags, to ensure that all participants understood the term. The total perception score ranged from 7 to 28. A high score indicates a high level of perception regarding household SWM. Participants’ practice of household SWM was assessed using 14 questions measured on a five-point Likert scale, where 1 denoted “never” and 5 denoted “always”. Four of the practice questions were reverse-coded: “I throw my household solid waste to drain”, “I throw my household solid waste to open dump”, “I throw my household solid waste to open field”, “I throw my household solid waste to the roadside”. For the question “I recycle or reuse solid waste at home”, the terms “recycling” and “reuse” were clarified, where necessary, using simple, context-relevant examples (e.g., reusing old clothes, or containers, and selling recyclable materials such as paper, or plastic to local vendors) during interviewer-administered data collection. The total practice score ranged from 14 to 70. To standardize the scores to the original five-point Likert scale, the total score was divided by 14, yielding a 1–5 scale. The mean of the converted scores was used as the cut-off point to categorize practice, a commonly used method in Knowledge, Attitudes, and Practices (KAP) surveys and composite score-based studies, where no standardized cut-off values exist [46,47]. Good practice of household SWM was considered if the participants scored higher than the mean score.

### Data analysis

Data were analysed using Stata version 18.0. Descriptive analyses were performed to report the frequencies and percentages of background characteristics, as well as the distribution of participants’ knowledge, perception, and practice regarding household SWM. Bivariate analyses were performed using Pearson’s chi-square test to observe the differences in the household SWM practice across different background characteristics. All independent variables including background characteristics, knowledge score and perception score were entered into the multivariable analysis. Binary logistic regression was performed to identify the predictors of good SWM practice among the participants. The adjusted odds ratios (AORs) with corresponding 95% confidence intervals (CIs) were reported. Considering p value < 0.05, the association were considered statistically significant to predict good SWM practice among the study participants.

### Ethical considerations

The study’s ethical clearance was obtained from the Institutional Review Board (IRB) of North South University (ethical approval reference number: 2025/OR-NSU/IRB/0203). Prior to data collection, a written informed consent was obtained from the participants. The purpose of the study, potential risk and benefit of participation, and the right to withdraw from the study at any time without any consequences were mentioned in the written consent and also explained to the participants verbally. Participants were also assured that the data would be used solely for research purposes while maintaining strict confidentiality and anonymity. The ethical principles outlined in the 1964 Declaration of Helsinki and its later amendments were followed to maintain ethical conduct throughout the study.

## Result

### Background information

The background characteristics of the study participants residing in DNCC and DSCC are presented in **Table 1**. The majority of the participants belonged to the age group of 26–35 years (42.74%), followed by the age group of 36–45 years (23.59%). Two-thirds (66.53%) of the participants lived in households comprising 4 or fewer members. Majority of the participants were highly educated, holding a bachelor’s degree or higher (67.34%). Regarding husbands’ education, a similar pattern was observed, with 73.19% of the participant’s husband also holding a bachelor’s degree or higher. More than half of the participants were employed (54.23%). Nearly two-thirds lived in rented houses (65.73%), while only 20.77% had a monthly household income above 100,000 BDT. Furthermore, 57.66% of the participants reported having no exposure to information regarding solid waste management.

**Table 1:** Distribution of background characteristics of the participants (N = 496).

Variable	Category	Frequency (n)	Percentage (%)
Age (year)	18-25	60	12.10
	26-35	212	42.74
	36-45	117	23.59
	≥ 46	107	21.57
Family size	≤ 4	330	66.53
	> 4	166	33.47
Highest education level	Secondary or below secondary	59	11.90
	Higher secondary	103	20.77
	Bachelor’s degree or higher	334	67.34
Husband’s highest education level	Secondary or below secondary	32	6.45
	Higher secondary	48	9.68
	Bachelor’s degree or higher	363	73.19
	Not applicable	53	10.69
Employment status	Not employed	227	45.77
	Employed	269	54.23
Monthly household income (BDT)	≤ 40,000	117	23.59
	41,000–70, 000	150	30.24
	71, 000–100,000	126	25.40
	> 100,000	103	20.77
Residential status	Own house or flat	170	34.27
	Rented	326	65.73
Exposure to SWM information	Yes	210	42.34
	No	286	57.66

### Participants’ knowledge of household solid waste management

The participants’ knowledge of household solid waste management (SWM) is presented in **Table 2**. About 65.73% of the participants did not know about the 3R concept (reduce, reuse, recycle) of waste management. However, the majority (85.48%) knew about organic waste, and 92.94% knew that it could be used as fertilizer. Although 18.55% of the participants did not consider solid waste a useful resource, the majority (86.09%) believed that it should be disposed of through proper sorting. Similarly, 86.49% knew that hazardous waste should be wrapped before disposal. While the majority of the participants recognized paper, plastic, metal, and cloth as recyclable and reusable waste (85.89%), a considerable percentage of participants did not know that recyclable wastes should be disposed of separately (17.54%) and that they should be kept dry (24.19%). A substantial proportion (41.73%) of the participants did not know that batteries, electronics, and paint were recyclable waste, and 34.48% were unaware that these items should be disposed of separately from other recyclable wastes.

**Table 2:** Participant’s knowledge of household solid waste management (N = 496).

Variable	Frequency (%)	
	Yes	No
Did you know about the 3R, the ideal way of waste management, before?	170 (34.27)	326 (65.73)
Do you know that different types of waste should be disposed of in different ways?	408 (82.26)	88 (17.74)
Solid waste can be utilized as a useful resource	404 (81.45)	92 (18.55)
Solid waste should be disposed of by proper sorting	427 (86.09)	69 (13.91)
Recyclable wastes are disposed of separately	409 (82.46)	87 (17.54)
Recyclable wastes should be kept dry	376 (75.81)	120 (24.19)
Paper, plastic, metal, and cloths are recyclable, and reusable waste	426 (85.89)	70 (14.11)
Batteries, electronics, and paint are recyclable waste	289 (58.27)	207 (41.73)
Batteries, electronics, and paint are disposed of separately from other recyclable waste	325 (65.52)	171 (34.48)
Hazardous waste should be wrapped before disposal	429 (86.49)	67 (13.51)
Do you know what organic waste is?	424 (85.48)	72 (14.52)
Organic waste can be used as fertilizer	461 (92.94)	35 (7.06)

### Participant’s perception towards household solid waste management

The participants’ perception towards household solid waste management is presented in **Table 3**. A total of 31.05% of the participants “strongly agreed” that purchasing products with minimal packaging could reduce the amount of waste, while only 18.55% “strongly agreed” that digitalization can reduce the amount of solid waste. A majority of the participants (64.52% “strongly agreed” and 32.06% “agreed”) believed that improper waste management can cause health problems. Similarly, 38.10% “strongly agreed” and 45.97% “agreed” that solid waste management is a problem in their area. Moreover, 64.11% of the participants “strongly agreed” that individuals in society should be part of a proper waste management system, while only 47.98% “strongly agreed” that sorting solid waste at the household level can lead to proper solid waste management in the city.

**Table 3:** Participant’s perception towards household solid waste management (N = 496).

Variable	Frequency (%)			
	Strongly disagree	Disagree	Agree	Strongly agree
Purchasing products with minimal packaging can reduce the amount of waste	9 (1.81)	64 (12.90)	269 (54.23)	154 (31.05)
Digitalization can reduce the amount of solid waste	32 (6.45)	132 (26.61)	240 (48.39)	92 (18.55)
Re-using the reusable products can reduce the solid waste	1 (0.20)	21 (4.23)	268 (54.03)	206 (41.53)
Improper waste management can cause health problems	6 (1.21)	11 (2.22)	159 (32.06)	320 (64.52)
Individuals from the society should be a part of proper waste management	5 (1.01)	7 (1.41)	166 (33.47)	318 (64.11)
Sorting solid waste at the household level can lead to proper solid waste management in the city	9 (1.81)	50 (10.08)	199 (40.12)	238 (47.98)
Solid waste management is a problem in your area	13 (2.62)	66 (13.31)	228 (45.97)	189 (38.10)

### Participant’s practice regarding household solid waste management

The participants’ practice regarding household solid waste management is presented in **Table 4**. More than half of the participants “always” disposed of solid waste regularly (54.64%), while only 14.92% “always” used different bins for different types of solid waste, and 61.29% “always” collected waste in a bin with a lid. Almost one-third of the participants “never” segregated biodegradable and non-biodegradable waste at home (34.48%). The majority “never” disposed of their household solid waste into drains (77.82%), open fields (89.72%) or roadsides (81.85%), while 9.48% “always” disposed of waste in open dumps. About 36.09% “always” reused grocery bags, and 17.94% “always” recycled kitchen waste as compost for gardening. Only 4.03% of the participants “always” recycled or reused solid waste at home. Similarly, only 10.69% “always” reused paper, metal, clothes, and plastic for household purposes, while 19.15% “always” recycled these items by selling them to a local vendor. About two-thirds of the participants “always” disposed of solid waste through waste collectors (63.51%).

**Table 4:** Participant’s practice regarding household solid waste management (N = 496).

Variable	Frequency (%)				
	Never	Seldom	Sometimes	Often	Always
I dispose solid waste regularly	7 (1.41)	32 (6.45)	87 (17.54)	99 (19.96)	271 (54.64)
I use different bins for different types of solid waste	222 (44.76)	82 (16.53)	61 (12.30)	57 (11.49)	74 (14.92)
I segregate biodegradable (paper, banana peels, cardboard and vegetables) and non-biodegradable (plastic, glass, steel, rubber) wastes at home	171 (34.48)	79 (15.93)	62 (12.50)	56 (11.29)	128 (25.81)
I throw my household solid waste to drain	386 (77.82)	48 (9.68)	35 (7.06)	12 (2.42)	15 (3.02)
I throw my household solid waste to open dump	317 (63.91)	53 (10.69)	55 (11.09)	24 (4.84)	47 (9.48)
I throw my household solid waste to open field	445 (89.72)	33 (6.65)	11 (2.22)	6 (1.21)	1 (0.20)
I throw my household solid waste to the roadside	406 (81.85)	46 (9.27)	35 (7.06)	6 (1.21)	3 (0.60)
I collect my household solid waste in a bin with a lid	78 (15.73)	35 (7.06)	27 (5.44)	52 (10.48)	304 (61.29)
I recycle or reuse solid waste at home	152 (30.65)	109 (21.98)	145 (29.23)	70 (14.11)	20 (4.03)
I reuse grocery bags	27 (5.44)	28 (5.65)	106 (21.37)	156 (31.45)	179 (36.09)
I recycle our kitchen waste (food waste) as compost for gardening	107 (21.57)	59 (11.90)	146 (29.44)	95 (19.15)	89 (17.94)
I reuse paper, metal, clothes, plastic for household purposes	80 (16.13)	100 (20.16)	144 (29.03)	119 (23.99)	53 (10.69)
I recycle paper, metal, clothes, plastic by selling them to a local vendor	75 (15.12)	73 (14.72)	144 (29.03)	109 (21.98)	95 (19.15)
I dispose solid waste to waste collectors	17 (3.43)	30 (6.05)	51 (10.28)	83 (16.73)	315 (63.51)

### Bivariate analysis of household solid waste management practice by background characteristics

The converted household SWM practice score ranged from 1 to 5, with a mean ± SD of 3.66 ± 0.47. Using this mean as the cut-off, 47.18% of participants were classified as having good household SWM practice.

**Table 5** presents the prevalence of good SWM practice by background characteristics of the participants. Good SWM practice was most prevalent among the age group of 18–25 years (53.33%), and the practice gradually declined with increasing age, with the lowest prevalence observed among the age group of ≥ 46 years (43.93%). However, the youngest age group (18–25 years) represented a smaller proportion of the sample compared with other age categories, which may affect the precision of the estimated prevalence for this group. Participants living in households comprising four or fewer members (47.88%) reported a higher prevalence of good SWM practice. A significantly higher prevalence of good SWM practice (p = 0.005) was observed among participants with a bachelor’s degree or higher (52.10%), followed by those with a higher secondary degree (39.81%) and those with a secondary or below secondary degree (32.20%), indicating that good practice increased with the level of education. Similarly, a higher prevalence of good practice was also observed among participants whose husbands had a bachelor’s degree or higher (49.31%). Participants who were unemployed reported a higher prevalence of good SWM practice (48.46%). Both monthly household income (p = 0.035) and residential status (p = 0.041) were significantly associated with good SWM practice. Participants with a monthly household income of 71, 000–100,000 BDT (57.94%) and those living in their own house or flat (53.53%) showed the highest prevalence of good SWM practice, whereas those with a monthly household income below 40,000 BDT (40.17%) and those living in rented homes (43.87%) reported the lowest prevalence. Furthermore, participants who had exposure to information regarding SWM (59.05%) had a significantly (p < 0.001) higher prevalence of good SWM practice compared to those without exposure (38.46%).

**Table 5:** Association between household solid waste management practice and by background characteristics (N = 496).

Variable	SWM Practice Frequency (%)		p-value
	Good	Bad	
Age (year)			
18-25	32 (53.33)	28 (46.67)	0.596
26-35	103 (48.58)	109 (51.42)	
36-45	52 (44.44)	65 (55.56)	
≥ 46	47 (43.93)	60 (56.07)	
Family size			
≤ 4	158 (47.88)	172 (52.12)	0.659
> 4	76 (45.78)	90 (54.22)	
Highest education level			
Secondary or below secondary	19 (32.20)	40 (67.80)	**0.005**
Higher secondary	41 (39.81)	62 (60.19)	
Bachelor’s degree or higher	174 (52.10)	160 (47.90)	
Husband’s highest education level			
Secondary or below secondary	12 (37.50)	20 (62.50)	0.119
Higher secondary	16 (33.33)	32 (66.67)	
Bachelor’s degree or higher	179 (49.31)	184 (50.69)	
Not applicable	27 (50.94)	26 (49.06)	
Employment status			
Not employed	110 (48.46)	117 (51.54)	0.600
Employed	124 (46.10)	145 (53.90)	
Monthly household income (BDT)			
≤ 40,000	47 (40.17)	70 (59.83)	**0.035**
41,000–70, 000	68 (45.33)	82 (54.67)	
71, 000–100,000	73 (57.94)	53 (42.06)	
> 100,000	46 (44.66)	57 (55.34)	
Residential status			
Own house or flat	91 (53.53)	79 (46.47)	**0.041**
Rented	143 (43.87)	183 (56.13)	
Exposure to SWM information			
No	110 (38.46)	176 (61.54)	**< 0.001**
Yes	124 (59.05)	86 (40.95)	

### Factors associated with household solid waste management practice

The overall knowledge score had a mean ± SD of 9.17 ± 2.46, while the overall perception score had a mean ± SD of 23.05 ± 2.87. Knowledge and perception scores were treated as continuous variables in the multivariable analysis. Before running the regression analysis, multicollinearity was assessed to determine if any of the independent variables were correlated or not. The variance inflation factor (VIF) values for all the independent variables were less than 5; therefore, all independent variables including background information, knowledge score and perception score were entered into the multivariable analysis.

**Table 6** presents the multivariable analysis of factors associated with household solid waste management (SWM) practice among residents of the two city corporations of Dhaka. Both knowledge and perception scores were significantly associated with increased odds of good SWM practice. Each one-point increase in the knowledge score was associated with a 23% increase in the odds of good SWM practice (AOR = 1.23; 95% CI: 1.12–1.35). Similarly, each one-point increase in the perception score was associated with a 14% increase in the odds of good SWM practice (AOR = 1.14; 95% CI: 1.06–1.22). Participants who had exposure to information regarding SWM had almost twice the odds of having good SWM practice compared to those without such exposure (AOR = 1.91; 95% CI: 1.27–2.89).

**Table 6:** Multivariable analysis of factors associated with SWM practice (N = 496).

Variable	AOR	95% CI	
Knowledge score	1.23	**1.12**	**1.35**
Perception score	1.14	**1.06**	**1.22**
Age (year)			
18-25	1.00 (Ref.)		
26-35	0.67	0.34	1.34
36-45	0.65	0.31	1.37
≥ 46	0.70	0.33	1.48
Family size			
≤ 4	1.00 (Ref.)		
> 4	0.86	0.57	1.30
Highest education level			
Secondary or below secondary	1.00 (Ref.)		
Higher secondary	1.52	0.65	3.56
Bachelor’s degree or higher	2.01	0.86	4.73
Husband’s highest education level			
Secondary or below secondary	1.00 (Ref.)		
Higher secondary	0.56	0.18	1.69
Bachelor’s degree or higher	0.61	0.22	1.73
Not applicable	0.64	0.20	2.02
Employment status			
Not employed	1.00 (Ref.)		
Employed	0.77	0.51	1.16
Monthly household income (BDT)			
≤ 40,000	1.00 (Ref.)		
41,000–70, 000	1.01	0.57	1.77
71, 000–100,000	1.45	0.79	2.65
> 100,000	0.70	0.35	1.38
Residential status			
Own house or flat	1.00 (Ref.)		
Rented	0.66	0.43	1.01
Exposure to SWM information			
No	1.00 (Ref.)		
Yes	1.91	**1.27**	**2.89**

## Discussion

Despite significant action taken by the two city corporations and the government, household solid waste management remains a pressing concern in Dhaka evident by scattered garbage and overflowing waste bins on the streets, clogged drains, open dumping on public spaces like streets or vacant plots. This study aimed to assess the prevalence of household SWM practice and identify its associated determinants among residents of the city corporations of Dhaka, Bangladesh. The study found the educational level of the participants, monthly household income, residential status, and exposure to solid waste management information to be significantly associated with SWM practice at the household level in bivariable analysis. However, in the multivariable analysis, only knowledge, perception, and exposure to SWM information remained significantly associated with household SWM practices. Education, income, and residential status variables were insignificant in the multivariable analysis, likely because they confound exposure to SWM information.

The current study found that household-level reuse of solid waste materials was relatively low compared to recycling practices. Contextual factors in Dhaka’s urban residential settings, including limited indoor space in rented apartments [20], widespread availability of disposable packaging [48], and access to unsegregated waste collection services [9], may discourage reuse. Consistent with another Bangladeshi study [32], recycling was found to be more commonly practiced than reuse because selling recyclable waste materials to local vendors provides small economic returns for households and is relatively easy, as vendors often collect recyclables door-to-door [18]. Low awareness of the 3Rs may further lead households to prioritize recycling over reuse, despite reuse being environmentally more sustainable [48]. In Bangladesh, where formal promotion campaigns for recycling and reuse remain limited, this awareness gap may contribute to the low prevalence of reuse behaviours observed in this study. Recycling kitchen waste as compost for gardening was also moderate. Previous research suggests that positive attitudes, better access to information, and practical familiarity with the composting process influence food waste composting intentions among home gardeners in Dhaka [49]. The study findings indicate that household waste segregation remained suboptimal, with more than one-third of participants reporting never segregating biodegradable and non-biodegradable waste at home. According to previous reports, a key reason for this low practice is that both the city corporations collect unsegregated waste, which may discourage households from sorting waste at the source [32]. The prevalence of bad household SWM practice in this study was 52.82%. These findings should be interpreted in the context of the background characteristics of the study participants. The majority of participants belonged to smaller households, which is consistent with the average household size in Urban Dhaka (approximately 3.8 persons) [20], suggesting reasonable comparability with the urban population structure. The high proportion of rented households observed in this study also aligns with existing urban housing patterns in Dhaka, where approximately 65–75% of residents live in rented accommodations [19]. In terms of educational attainment, only about 15% of the overall population of Dhaka have graduate-level education [50], which is considerably lower than that observed in our sample. This difference may be attributed to the study setting, as the sample was drawn from areas under the Dhaka North and Dhaka South City Corporations, which represent predominantly urban populations with comparatively higher educational attainment. The presence of bad SWM practices even among a relatively educated urban population suggests that structural urban barriers, such as limited household space [20] and access to unsegregated door-to-door waste collection services [8], as well as individual factors like ignorance or unwillingness [32], may influence SWM practices alongside individual knowledge and perception.

More than half of the study participants (52.82%) were found to have bad practice of household solid waste management (SWM). The prevalence was found to be lower than the previous studies conducted in Ethiopia [25–28,51–53], Uganda [54] and Ghana [27] but higher than the study conducted in Kenya [29]. Differences in the sociodemographic and socioeconomic status, lifestyle, geographic area, weak enforcement of existing policies and the infrastructure of the solid waste management system might explain the variations in the prevalence [27,51,52] observed between the studies.

Participants with higher knowledge on household solid waste management were found to have higher odds of good solid waste management practices. The positive association suggests that even a slight increase in residents’ knowledge, such as correctly answering one additional knowledge question, increases the odds of practicing good SWM by 23%. This underscores the potential role of knowledge in shaping household SWM practices. The finding is consistent with studies conducted in Ethiopia and Thailand, where knowledge regarding SWM was significantly associated with good practices [22,25–28]. A positive relationship where increased knowledge supports the correct implementation of waste management practices could explain this result [22,27]. Moreover, individuals with poor knowledge of SWM tend to have poor sense of environmental hygiene, which may lead to poorer practice of household SWM [27,55]. In contrast, Almasi et al. [56] and Zand et al. [57] reported that adequate knowledge did not result in proper SWM practice. However, the significance of their results was not established through multivariable analysis; rather it was based on bivariable analysis without controlling for other covariates. Thus, their contradictory findings are not strong enough to challenge the findings of the present study. Although the discrepancy across studies could also be attributed to differences in awareness levels regarding health, limited resources, inadequate involvement of community, poor infrastructure, or inconsistent recycling initiatives by the local authority [25,56].

The present study found that participants with a good perception of solid waste management had higher odds of having good SWM practice. The positive association indicates that even a slight increase in perception, such as correctly answering one additional perception question, increases the odds of practicing good SWM by 14%. A probable reason behind this finding could be that people’s perception reflects their opinions as well as their behaviour toward proper solid waste management [32]. The findings align with studies conducted in Bangladesh, Ghana, and Malaysia who have highlighted a positive relationship between people’s perception and practice regarding SWM [24,29,32]. Evidence from different studies showed people who have a perception that improper waste management is a reason behind disease manifestation were more likely to exhibit good SWM practice [24,29]. Cleanliness could be another factor that people perceive for their motivation to proper waste management [24,29]. A study conducted by Yoada et al. [29] highlighted the fact that people’s perception towards solid waste management was fairly low so it was unlikely that their waste management practices would be better.

Exposure to SWM information was found to be a highly significant factor in determining the good or bad SWM practice among the study participants both in bivariable and multivariable analysis. Participants with exposure to SWM information were found to have higher odds of good SWM practice compared to their counterparts. Similar findings were reported in different studies where participants who did not receive information regarding waste management showed low levels of SWM practice compared to those who received information through different types of mediums [22,23,52]. Another study on solid waste management highlighted the importance of exposure to SWM information through training or education by means of various channels for good practice of SWM [23]. Having access to information regarding SWM through different mediums can significantly enhance community awareness and positively influence waste-sorting behaviour, consequently leading to good SWM practices [23].

In addition, education level, monthly household income, and residential status were found to be significant in the bivariate analysis. The results from the bivariate analysis showed that participants with higher educational level practice good solid waste management compared to the participants with lower educational level. Our findings are similar to those studies carried out in Ethiopia [51–53,58] and Sabah [59] who also reported a positive association between education level and SWM practice. A significant cluster of people who belong to the high educational background come with better knowledge and are more inclined towards household waste separation and show higher willingness to adopt waste reduction and recycling practices ultimately leading to good SWM practice [57,60]. Another reasonable explanation behind this finding could be that people who belong to the educated class exhibit better knowledge and awareness on the environmental and health impacts of waste and that reusing and recycling waste can significantly help reduce environmental pollution [61]. Extensive research has established a positive association between education level and SWM practices. However, a study carried out by Wang et al. [58] reported that people with lower educational levels were more willing to take part in household waste recycling and thus actively taking part in such activities. Challenges related to solid waste management can be addressed through such economic approaches, which in turn foster sustainable development and public health outcomes [52]. This finding somewhat contradicts the widely held belief that higher education invariably results in good solid waste management practices [54].

In the bivariate analysis, practice was found significantly good among the participants who belong to a household with high monthly income compared to those belonging to the lower-income group. Similar results were observed in studies where monthly income of the household was significantly associated with household SWM practice [52,53,62]. The positive association between low-income households and bad SWM practices could be explained by the reality that such households buy inexpensive products which are less durable leading to increased waste generation [52]. Similar to findings from other urban areas, low-income households of urban Dhaka, which is under the coverage of the two city corporations, often face financial constraints resulting in insufficient waste storage bins and limited space for multiple bins for waste sorting and storage, which may discourage good SWM practices [32,63]. It has been statistically established by Noufal and Malla [64] that household income increases the willingness to separate household waste leading to good SWM practice. Moreover, households with high income had greater access to a wider range of media, thus they are more enlightened about environmental issues and to appreciate the environmental and social merits of proper solid waste disposal [64,65]. Contradicted results were established by Alhassan et al. [65] who found that households with a higher income were less inclined towards practicing waste separation as affluent people are busy with their work and can’t spare time and effort from their busy schedules to manage their household solid waste properly. Taye et al. [66] also found similar results where people belonging to the high-income levels had strong inclination towards open space waste disposing practices. A possible explanation for this bad practice of waste management could be high income households generate greater amounts of household solid waste possibly due to higher consumption levels [66].

People living in their own household were found to have significantly good SWM practice compared to the residents of the rented household in the bivariate analysis. A study conducted in Syria also found a positive relationship between residence type and waste related behaviour where families living in a residential property were more likely to practice waste separation at source [64]. Homeowners showed more concern and greater enthusiasm to conserve the cleanliness of the surroundings where else rentals cared less about it [64]. This attitude of rentals could be due to the absence of a feeling of belonging to the place as they are temporarily living over there [64]. Another possible explanation is that many rented households in urban Dhaka are compact with limited space [19], and smaller sizes [20], which may reduce the feasibility of waste segregation, storage, reuse, and recycle within households. Contrary to the findings of the present study, Genati et al. [51] found that rentals managed solid waste properly compared to private house owners. This finding can be explained by the fact that houseowners keep the tenants under their close supervision and the tenants are held accountable for their actions [51].

### Strength and Limitations of the Study

The strength of this study lies in its novelty, as, to the researchers’ knowledge, it is the first to investigate the association of knowledge and perception of household solid waste management, along with other associated factors, with practice among residents of the city corporations of Dhaka, using a relatively large sample (N = 496). The study has used a comprehensive measurement of household solid waste management practice incorporating concepts like waste disposal behaviour, waste separation, 3R behaviour (reduce, reuse, recycle), usage of door-to-door waste collection services, making the study outcome more robust.

However, the study is not without limitations, as no causal inferences could be made between the independent variables and the outcome variable due to the cross-sectional design of the study. The study included participants living in formal residential buildings within the areas of DNCC and DSCC, excluding slum areas that also fall under the coverage of both city corporations. The study focused only on women who were primarily responsible for household SWM practices; thus, men were excluded from the sample, as well as those women who were living alone without families. The exclusion of slums, the convenient selection of study participants, limits the generalizability of the findings to all households in the city corporations of Dhaka.

### Recommendations

1. Community-based structured awareness campaigns on household SWM practices should be implemented jointly by DNCC and DSCC in collaboration with public health professionals to increase the public exposure to SWM-related information. Priority should be given to lower-income and less-educated households, as they are more likely to have limited exposure to such information.
2. More exposure to SWM-related information, such as practical guidance on proper household SWM practices, including waste sorting, segregation, disposal methods, and the principles of the 3Rs (reduce, reuse, and recycle), should be enhanced through multiple communication channels like social media, television, newspapers, posters, flyers, and local government announcements.
3. Public dissemination of existing national rules and strategies, including the Solid Waste Management Rules, 2021, and the National 3R Strategy for Waste Management (2010), should be strengthened by DNCC and DSCC through mass media, printed materials such as leaflets and posters, social media platforms, and local government-led awareness campaigns to enhance public exposure to SWM-related information.

## Conclusion

The findings of the current study revealed that household solid waste management (SWM) practices in DNCC and DSCC remain suboptimal, with less than half of the participants demonstrating good practice. Households also showed limited engagement in reduce, reuse, and recycling (3R) behaviours. Knowledge, perception, and exposure to SWM-related information emerged as significant determinants of household practices, highlighting the need for effective communication strategies. Future research may explore communication strategies to effectively promote proper household SWM practices.
